# Seasonal Trends and Antibiotic Resistance Profiles of Bacterial Pathogens in Indian Clinical Isolates

**DOI:** 10.7759/cureus.77255

**Published:** 2025-01-10

**Authors:** Nirmala B., Vanya Singh, Balram J Omar

**Affiliations:** 1 Microbiology, All India Institute of Medical Sciences, Rishikesh, Rishikesh, IND

**Keywords:** antimicrobial resistance, bacteria, diseases, seasonal trends, temperature

## Abstract

Introduction

Bacterial diseases exhibit seasonal trends, necessitating their monitoring for outbreak prediction, treatment optimization, and infection control. This study explores seasonal trends, temperature correlations, and antimicrobial resistance profiles of key pathogens in an Indian tertiary care setting.

Methodology

This cross-sectional study analyzed bacterial isolates from 1,562 patient samples, including *Staphylococcus aureus*, *Klebsiella pneumoniae*, *Pseudomonas aeruginosa*, *Acinetobacter baumannii*, *Escherichia coli*, and *Enterococcus faecalis*. Monthly infection rates and seasonal patterns were visualized using heatmaps and time-series graphs. Pearson’s correlation assessed the relationship between these infection rates and temperature. Antibiotic susceptibility was evaluated using VITEK2, with resistance patterns visualized in R.

Results

Infections peaked in April (*n* = 163, 10.43%) and March (*n* = 161, 10.30%), with *S. aureus* as the most common pathogen (*n* = 271, 25.64%), followed by *K. pneumoniae* (*n* = 201, 19.02%) and *P. aeruginosa *(*n* = 178, 16.84%). Seasonal trends showed *S. aureus* infections peaked in summer (*n* = 45, 16.6%), while *P. aeruginosa *(*n* = 27, 15.2%) and *E. faecalis *(*n* = 24, 25.5%) peaked in winter. Temperature correlated positively with *S. aureus* infection (*r* = 0.814, *P* = 0.001) and negatively with *P. aeruginosa* (*r* = -0.845, *P* = 0.001), and *E. faecalis *(*r* = -0.618, *P* = 0.032), with no correlation observed for *K. pneumoniae*, *A. baumannii*, and *E. coli*. Multi-drug resistance (MDR), extensively drug resistance (XDR), and pandrug resistance were more prevalent in Gram-negative than in Gram-positive bacteria.

Conclusions

This study reveals temperature-driven seasonal patterns in bacterial infections, aiding outbreak prediction and prevention. The findings emphasize the threat of multidrug resistance, particularly in Gram-negative bacteria, reinforcing the need for enhanced infection control and targeted antibiotic stewardship.

## Introduction

Disease from a historical perspective was often recognized as a disruption of the body’s natural state, believed to be caused by supernatural forces before the scientific era [1]. It wasn’t until the development of germ theory in the 19th century that microorganisms were identified as the cause of diseases [2]. Today, bacterial infections remain a significant public health concern worldwide, contributing to morbidity and mortality in both hospital and community settings [3]. The foundation of our understanding of bacterial diseases began in 1876 when German Physician Robert Koch established the direct link between bacteria and disease by identifying *Bacillus anthracis *as the causative agent of anthrax [4], which marked the beginning of bacteriology and set the stage for future discoveries.

However, moving to the 20th century, antibiotics emerged as a powerful weapon against bacterial infections. The discovery of penicillin by Alexander Flemming in 1928, and its widespread application during World War II revolutionized the treatment of bacterial diseases [5]. Nevertheless, this advancement also paved the way for the emergence of multi-drug-resistant (MDR) bacteria, largely due to the overuse of antibiotics [6]. This growing resistance has become a significant concern in recent years, complicating treatment options and increasing healthcare costs [7]. The World Health Organization (WHO) has recognized antimicrobial resistance (AMR) as one of the top public health threats globally, as it diminishes the efficacy of antibiotics, leading to more severe infections, prolonged hospital stays, and increased mortality. AMR is particularly concerning for hospital-associated pathogens that frequently develop resistance to multiple drug classes, highlighting the urgent need for continued surveillance and updated treatment guidelines [8].

India’s Ministry of Health and Family Welfare launched the National Action Plan for Antimicrobial Resistance to enhance awareness, improve surveillance, and strengthen infection prevention and control measures [9]. Recent research has revealed significant discoveries of new antibiotic resistance mechanisms in India, including the identification of New Delhi metallo-beta-lactamases in Gram-negative bacteria, AmpC-mediated resistance in Enterobacteriaceae, the emergence of vancomycin-resistant *Enterococci*, and extensively drug-resistant (XDR) *Mycobacterium tuberculosis*. Additionally, resistance to crucial last-resort antibiotics, such as carbapenems and colistin in Gram-negative bacteria, poses a serious challenge to treatment options [9].

Many of these infections exhibit seasonal patterns influenced by environmental factors such as temperature and humidity [10]. Previous research has demonstrated that these factors significantly affect the prevalence of specific pathogens. For instance, certain bacterial infections, like Gram-negative bacteria, tend to peak during warmer months [11], while others, such as *Streptococcus pyogenes*, may surge in colder periods due to variations in human behavior, increased exposure to vectors, or changes in the bacterial life cycle [12]. This is supported by a study by Paireau et al., which reported that the incidence of various bacterial meningitis cases, including those caused by *Neisseria meningitis*, *Haemophilus influenzae*, and *Streptococcus pneumoniae*, showed seasonal trends globally [13].

Seasonal trends in bacterial infections emphasize the critical need for continuous monitoring to predict outbreaks accurately, allocate healthcare resources efficiently, and implement focused prevention strategies. Such information can guide public health policies and clinical practices, ultimately enhancing our ability to respond proactively to bacterial diseases [14]. For instance, understanding winter peaks in respiratory infections like strep throat allows for timely interventions, such as vaccination campaigns and increased clinical preparedness. Recognizing these seasonal epidemiological patterns is crucial for effective infection control and prevention strategies, shaping a responsive healthcare system that adapts to seasonal risks [15].

This study focuses on six prevalent bacterial pathogens: *Staphylococcus aureus*, *Klebsiella pneumoniae*, *Pseudomonas aeruginosa*, *Escherichia coli*, *Acinetobacter baumannii*, and *Enterococcus faecalis*, which are frequently linked to healthcare-associated infections. Known for their ability to develop MDR, these organisms present significant treatment challenges for clinicians [9,16].

This study collected data from 1,562 clinical samples, to identify bacterial species, conduct antibiotic susceptibility testing, and perform geospatial analysis of patient samples to understand the regional infection patterns. Seasonal trends and their correlations with environmental factors, such as temperature, were also analyzed. This study aims to enhance our understanding of bacterial infection epidemiology in North India, emphasizing the need for ongoing surveillance to monitor antibiotic resistance trends and guide treatment protocols.

## Materials and methods

Setting and patients

**Figure 1:**
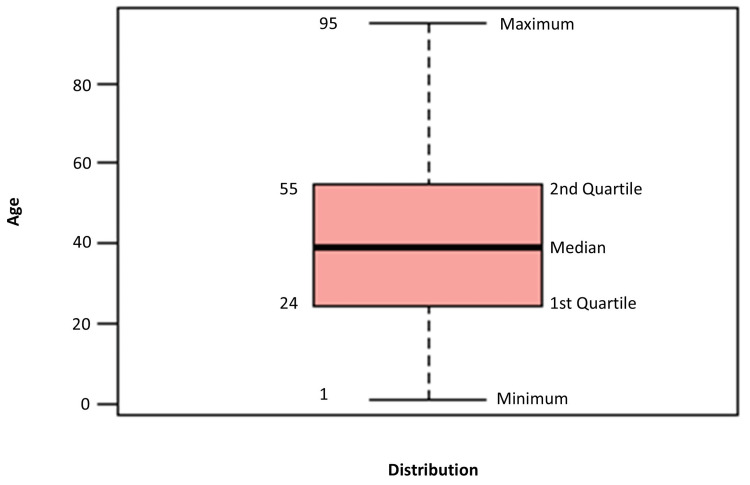
Distribution of patient ages in the dataset, highlighting the median and interquartile range (IQR) values.

This cross-sectional, observational study analyzed bacterial isolates from 1,562 patient samples collected between 2023 and 2024 at the Bacteriology Laboratory, All India Institute of Medical Sciences (AIIMS), Rishikesh. The patients' ages ranged from 1 to 95 years, with a median age of 39 years (Figure 1). Ethical clearance was obtained from the Institutional Ethics Committee under Letter No. AIIMS/IEC/23/294.

Bacterial isolation and identification

Clinical specimens, including blood, urine, sputum, tissue samples, aspirates, pus, bronchoalveolar lavage (BAL), cerebrospinal fluid (CSF), and other body fluids, were processed using standard microbiological techniques. Initial isolation involved culturing the samples on appropriate media, such as blood agar, MacConkey agar, and Cystine-Lactose-Electrolyte-Deficient (CLED) agar, followed by incubation at 37 °C. Bacterial species were identified through colony morphology, Gram staining, and biochemical tests (such as catalase, coagulase, and oxidase).

Furthermore, bacterial identity (ID) confirmation and antibiotic susceptibility testing (AST) were conducted using the VITEK 2 automated system (bioMérieux, Lyon, France), processing individual bacterial isolates with the relevant VITEK 2 cards - ID cards for bacterial identification and AST cards for susceptibility testing.

Definitions

According to the European Centre for Disease Prevention and Control (ECDC) and the Centers for Disease Control and Prevention (CDC), MDR is defined as acquired resistance to at least one agent in three or more antimicrobial classes. Additionally, XDR refers to acquired resistance to at least one agent in all antimicrobial classes except for two or fewer. Finally, pandrug resistance (PDR) is acquired resistance to all agents in all antimicrobial classes [17].

Data analysis

Data were stored in Microsoft Excel for initial processing. To visualize the geographical distribution of patient samples, we used ArcGIS software, allowing us to map sample locations. Next, we calculated and plotted disease rates for each month using Microsoft Power BI, providing insights into trends over time.

Pathogens were selected based on their isolation frequency from clinical samples over one year. We included only those bacterial species identified in a significant number of cases, excluding those with fewer cases and contaminants. The top six bacterial species with the highest counts were selected for further analysis.

To assess phylogenetic relationships, we constructed a tree of the top six bacterial species using the PhyloT tool and visualized using the Interactive Tree of Life (iTOL) platform (Biobyte Solutions GmbH, Heidelberg, Germany), employing National Center for Biotechnology Information (NCBI) taxonomy IDs for accurate classification.

To analyze the seasonal patterns of individual bacteria, we created heatmaps and seasonal time series graphs using the forecast package in R software (R Foundation for Statistical Computing, Vienna, Austria). Pearson's correlation coefficient was calculated using IBM SPSS version 21 (IBM Corp., Armonk, NY), with statistical significance set at *P* < 0.05 to evaluate the relationship between temperature and infection rates. For AST, 100 random clinical isolates were selected from each bacterial species, and the results were presented using heatmaps to display resistance, intermediate, and susceptible (R/I/S) classifications.

## Results

Monthly distribution of bacterial disease counts

**Figure 2:**
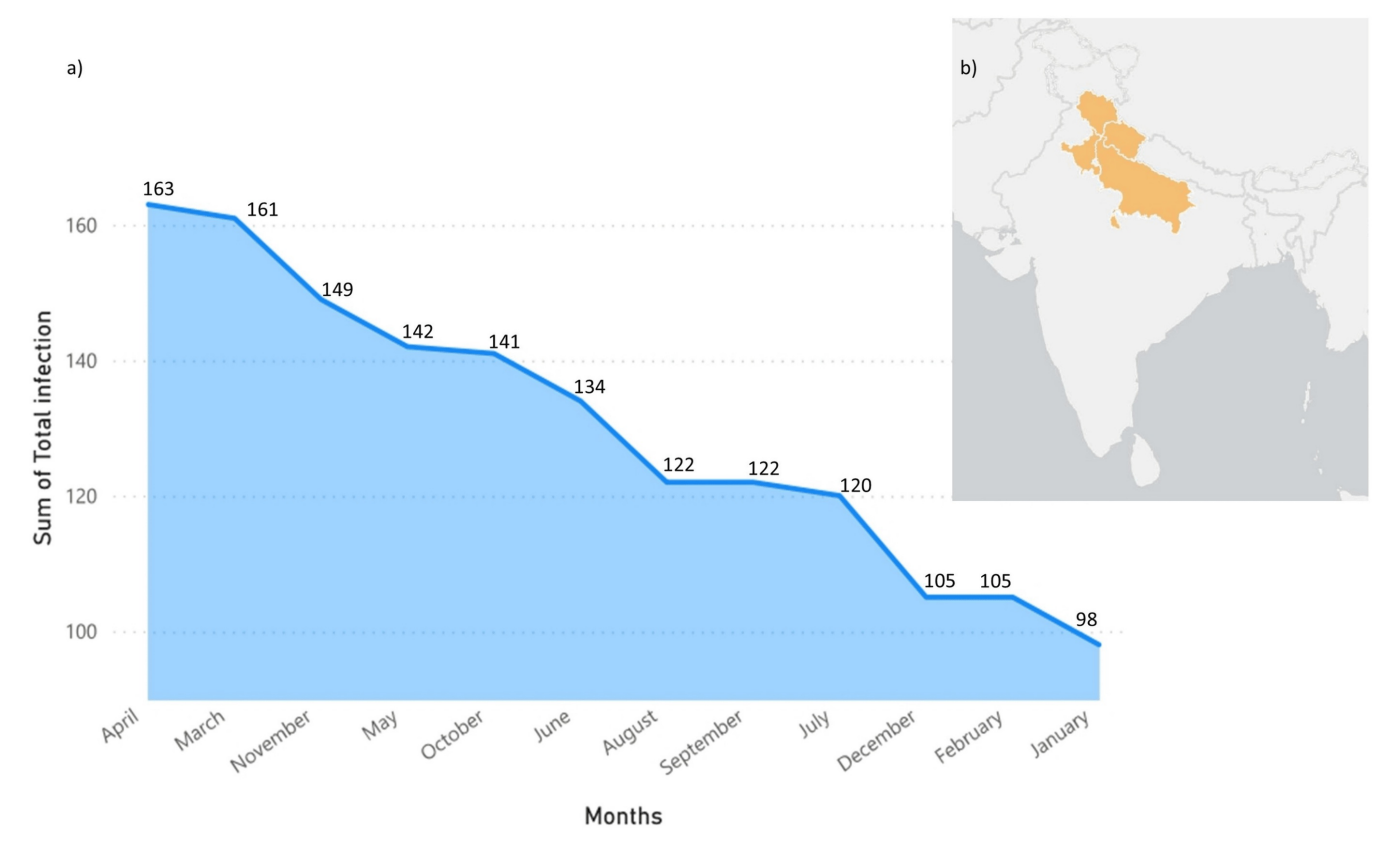
(a) The monthly distribution of bacterial disease counts; (b) an ArcGIS map illustrating the geographical locations of patient samples across North India.

Figure 2 illustrates the monthly distribution of bacterial disease counts from our study conducted in India, with the geographical context depicted on the ArcGIS map. Our analysis revealed significant fluctuations in infection rates throughout the year, with notable peaks in bacterial diseases occurring in April (*n* = 163, 10.43%) and March (*n* = 161, 10.30%), with the highest infection rates recorded. In contrast, infection rates were lowest in January (*n* = 98, 6.28%), February (*n* = 105, 6.72%), and December (*n* = 105, 6.72%).

Phylogenetic Analysis

**Figure 3:**
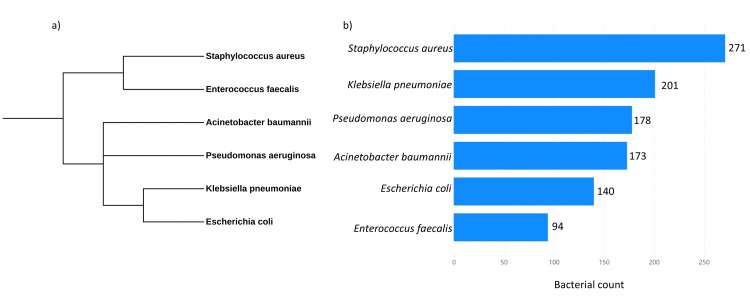
(a) A phylogenetic tree of the six most prevalent bacterial species, constructed using the Phylot tool (Interactive Tree of Life [iTOL] platform, Biobyte Solutions GmbH, Heidelberg, Germany), employing NCBI taxonomy IDs for precise classification; (b) distribution counts of each of the top six bacterial species. NCBI, National Center for Biotechnology Information

A phylogenetic tree was constructed for the top six bacterial species identified in the dataset (Figure 3). The analysis revealed that *S. aureus* was the most prevalent species, with 271 cases (25.64%), followed by *K. pneumoniae* with 201 cases (19.02%). *P. aeruginosa* accounted for 178 cases (16.84%), while *A. baumannii* had 173 cases (16.37%). *E. coli* was present in 140 cases (13.25%), and *E. faecalis* had the lowest count at 94 cases (8.89%). Additionally, other species found in fewer numbers included *Elizabethkingia meningoseptica*, *Enterobacter cloacae*, *Stenotrophomonas maltophilia*, *Burkholderia cepacia*, *Salmonella *sp., *Streptococcus* sp., *Proteus mirabilis*, and *Serratia marcescens*. The tree highlights the evolutionary relationships between these dominant pathogens, complementing the bacterial prevalence data.

Infection patterns and seasonal trends

**Figure 4:**
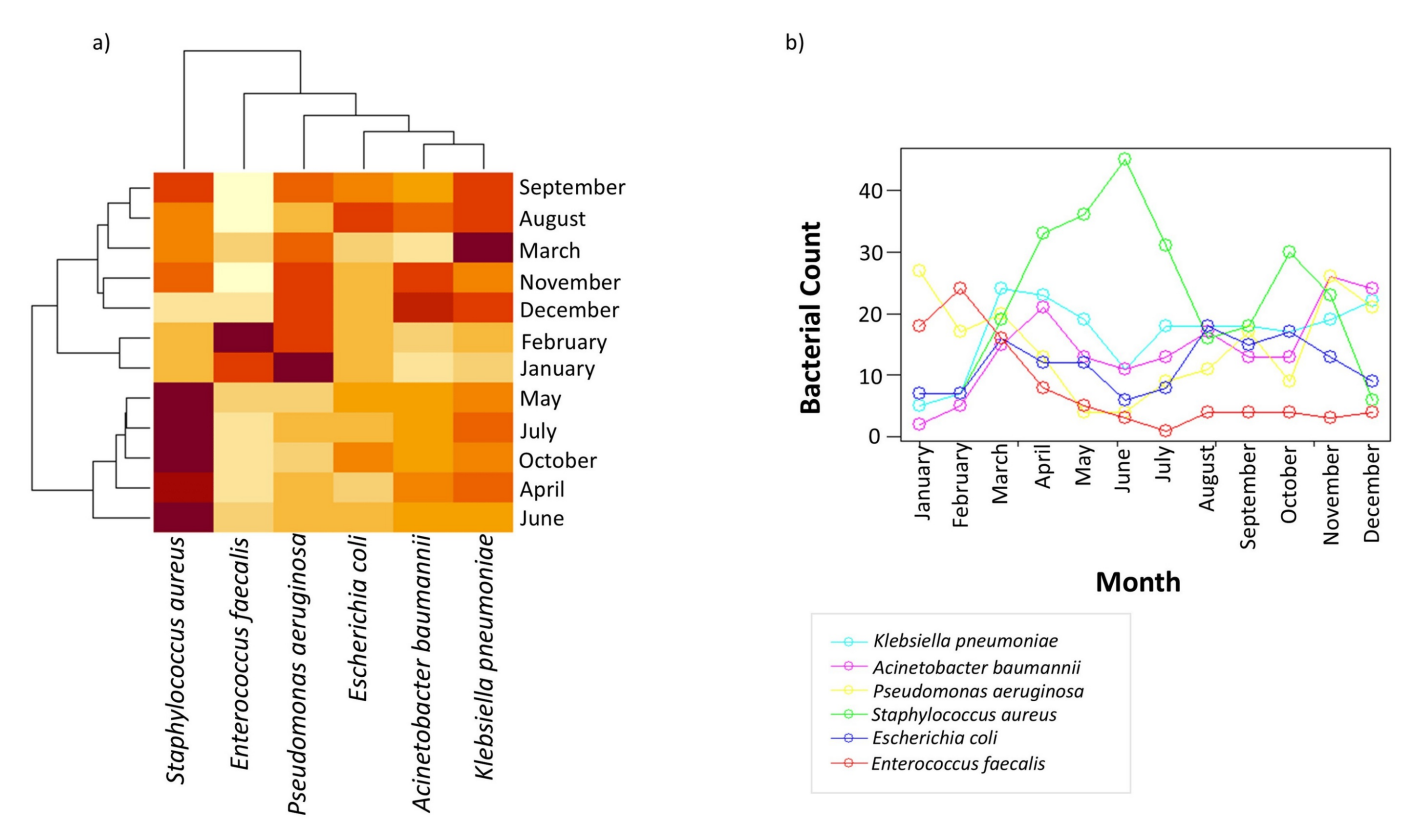
(a) A heatmap displaying the distribution patterns of individual bacterial species across each month, where darker colors represent a higher number of cases; (b) a seasonal time series graph generated using the forecast package in R software (R Foundation for Statistical Computing, Vienna, Austria), depicting trends of individual bacterial species across all months.

We created a heatmap to illustrate the monthly infection patterns for each bacterial species, with darker colors representing higher infection rates and lighter colors indicating lower rates. To further enhance our understanding of these infection patterns, seasonal time series graphs were developed for each species, to display their trends over the months. The graph revealed distinct patterns: *S. aureus* peaked in June (*n* = 45, 16.6%), with a secondary rise in October (*n* = 30, 11.1%), *E. faecalis* in February (*n* = 24, 25.5%), and *P. aeruginosa* in January (*n* = 27, 15.2%) with a secondary peak in November (*n* = 26, 14.6%). In contrast, *E. coli*, *A. baumannii*, and *K. pneumoniae* maintained consistent infection rates across all months, confirming the uniform pattern observed in the heatmap (Figure 4).

Temperature correlation with infection rates

Temperature data for all months were correlated with bacterial counts using Pearson correlation analysis (two-tailed). Our findings revealed a strong negative correlation between *P. aeruginosa* infection rate and temperature (*r* = -0.845, *P* = 0.001), suggesting that higher temperatures are associated with fewer infections. A moderate negative correlation was also observed for *E. faecalis* infection rate and temperature (*r* = -0.618, *P* = 0.032), both statistically significant. In contrast, a strong positive correlation was identified for *S. aureus* (*r* = 0.814, *P* = 0.001), indicating that rising temperatures significantly increase the incidence of *S. aureus *infections. However, no statistically significant correlations were found for *K. pneumoniae *(*r *= 0.325, *P *= 0.302), *A. baumannii* (*r* = 0.105, *P* = 0.744), and *E. coli* (*r* = 0.255, *P* = 0.424), as their *P*-values were >0.05.

Antibiotic susceptibility patterns

**Figure 5:**
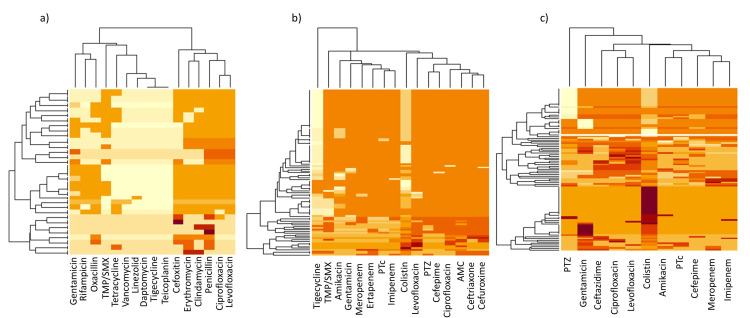
A heatmap of antimicrobial resistance patterns across various antibiotics, with darker colors indicating resistance and lighter colors indicating sensitivity for (a) Staphylococcus aureus, (b) Klebsiella pneumoniae, and (c) Pseudomonas aeruginosa.

**Figure 6:**
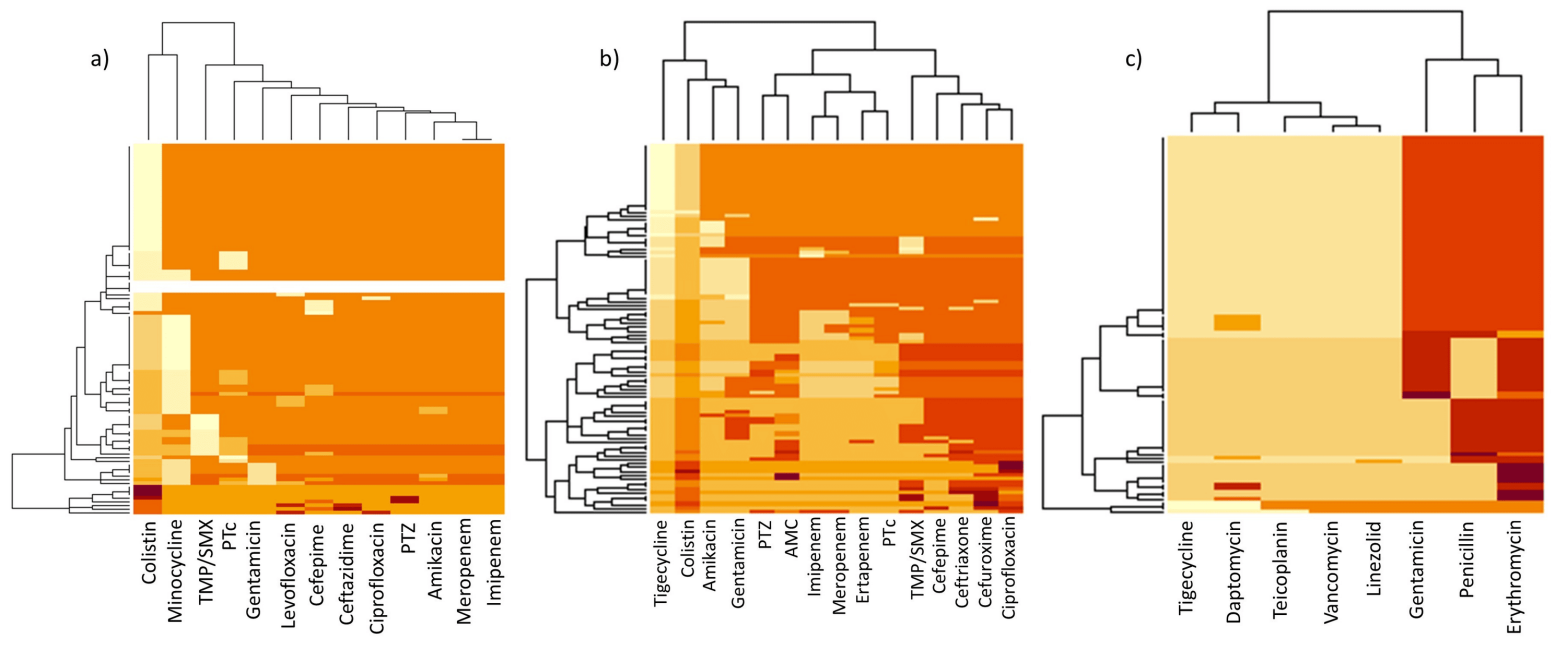
A heatmap of antimicrobial resistance patterns across various antibiotics, with darker colors indicating resistance and lighter colors indicating sensitivity for (a) Acinetobacter baumannii, (b) Escherichia coli, and (c) Enterococcus faecalis.

For antibiotic susceptibility testing, 100 random clinical isolates were selected from each bacterial species. The heatmap displays the antibiotic susceptibility patterns for *S. aureus*, *K. pneumoniae*, *P. aeruginosa*, *A. baumannii*, *E. coli*, and *E. faecalis* (Figures 5, 6). This visualization highlights each bacterium’s resistance and susceptibility patterns to various antibiotics, providing a comprehensive view of their antibiotic response profiles.

The resistance profile of *S. aureus* reveals significant resistance across several antibiotic classes. Within the beta-lactam group, resistance was observed to penicillin (*n* = 93, 93%), cefoxitin (*n* = 65, 65%), and oxacillin (*n *= 51, 51%). Both macrolides and lincosamides showed high resistance levels, with erythromycin and clindamycin showing 72% resistance (*n* = 72). For fluoroquinolones, resistance to ciprofloxacin (*n* = 72, 72%) and levofloxacin (*n* = 75, 75%) was recorded, while 39% of isolates (*n* = 39) were resistant to the aminoglycoside gentamicin. Resistance was further observed for rifampicin (*n* = 30, 30%) from the ansamycin class and trimethoprim/sulfamethoxazole (*n* = 44, 44%), a folate pathway inhibitor. Although lower, emerging resistance to tetracycline (*n* = 25, 25%) and glycopeptide vancomycin (*n* =3, 3%) was also detected. Notably, no resistance (*n* = 0, 0%) was observed against daptomycin (lipopeptides), tigecycline (glycylcyclines), linezolid (oxazolidinones), or teicoplanin (glycopeptides), indicating these antibiotics remain effective options (Figure 5).

*K. pneumoniae* isolates demonstrated extensive resistance across multiple antibiotic classes. In the beta-lactam category, high resistance rates were noted for cefoperazone-sulbactam (PTc) (*n* = 75, 75%). Aminoglycosides also showed substantial resistance, with 56% of isolates (*n* = 56) resistant to amikacin and 74% (*n* = 74) to gentamicin. Among fluoroquinolones, levofloxacin and ciprofloxacin were highly resistant at 89% (*n* = 89) and 87% (*n* = 87), respectively, and 76% of isolates (*n* = 76) were resistant to trimethoprim/sulfamethoxazole in the sulfonamide class. Notably, resistance to tigecycline, a last-resort antibiotic was found in 13% of cases (*n* = 13), and colistin (polymyxin class) resistance rates were 14% resistant (*n* = 14) and 86% intermediate (*n* = 86) (Figure 5).

*P. aeruginosa* exhibited resistance across multiple antibiotic classes. In the beta-lactam group, resistance rates were notable for ceftazidime (*n* = 51, 51%), meropenem (*n* = 47, 47%), imipenem (*n* = 46, 46%), cefoperazone-sulbactam (*n* = 41, 41%), cefepime (*n* = 34, 34%), and piperacillin-tazobactam (PTZ, *n* = 18, 18%). Among aminoglycosides, gentamicin and amikacin showed 48% (*n* = 48) and 39% (*n* = 39) resistance rates, respectively. Fluoroquinolone resistance was also high, with ciprofloxacin at 53% (*n* = 53) and levofloxacin at 55% (*n* = 55). Notably, colistin, a last-resort polymyxin antibiotic, showed 12% resistance (*n* = 12), with 88% of isolates (*n* = 88) classified as intermediate (Figure 5).

In evaluating *A. baumannii*, most of the tested bacterial isolates resisted a diverse array of antibiotics. In the sulfonamide class, trimethoprim/sulfamethoxazole showed 81% resistance (*n* = 81). Among beta-lactams, resistance rates were particularly high, including cefoperazone-sulbactam (*n* = 73, 73%), cefepime (*n* = 82, 82%), ceftazidime (*n* = 93, 93%), PTZ (*n* = 92, 92%), meropenem (*n* = 91, 91%), and imipenem (*n* = 91, 91%). Aminoglycosides were also largely ineffective, with resistance to gentamicin at 85% (*n* = 85) and amikacin at 87% (*n* = 87). Similarly, high resistance rates were seen in fluoroquinolones with levofloxacin and ciprofloxacin showing 88% (*n* = 88) and 91% (*n* = 91) resistance, respectively. Colistin, a last-resort polymyxin antibiotic, exhibited 4% (*n* = 4) resistance, with 96% of isolates (*n* = 96) showing intermediate susceptibility. Additionally, 50% of isolates (*n* = 50) demonstrated resistance to minocycline, indicating substantial challenges for tetracycline-class antibiotics (Figure 6).

*E. coli *isolates exhibited high resistance across multiple antibiotic classes. Among beta-lactams, high resistance rates were observed with cefuroxime (*n* = 92, 92%), ceftriaxone (*n* = 89, 89%), cefepime (*n* = 82, 82%), amoxicillin-clavulanate (*n* = 70, 70%), and PTZ (*n* = 68, 68%). Fluoroquinolone resistance was particularly significant, with 92% of isolates (*n* = 92) resistant to ciprofloxacin. Sulfonamide resistance, notably to trimethoprim/sulfamethoxazole, was also high (*n* = 72, 72%). Carbapenems demonstrated moderate resistance, with ertapenem (*n* = 50, 50%), meropenem (*n* = 48, 48%), and imipenem (*n* = 43, 43%). Aminoglycosides gentamicin and amikacin showed 43% (*n* = 43) and 25% (*n* = 25) resistance, respectively. Colistin exhibited intermediate susceptibility in all isolates, while tigecycline, a last-resort antibiotic, showed no resistance (*n* = 0, 0%) (Figure 6).

Finally, *E. faecalis* exhibited high resistance to antibiotics across several classes. In the macrolide class, erythromycin resistance was notably high at 89%, and within beta-lactams, penicillin demonstrated a 74% resistance rate (*n* = 74). Significant resistance was also noted to the aminoglycoside gentamicin (*n* = 73, 73%). However, resistance to the glycopeptides teicoplanin and vancomycin was low, at 3% (*n* = 3) and 4% (*n* = 4), respectively. The oxazolidinone linezolid also showed low resistance at 4% (*n* = 4), while no resistance was observed against tigecycline (*n* = 0, 0%), a last-resort glycylcycline, and lipopeptide daptomycin (*n* = 0, 0%) (Figure 6).

## Discussion

Bacterial infections continue to be a significant public health issue, especially in hospital settings where MDR pathogens are prevalent. Monitoring seasonal infection trends is crucial for predicting outbreaks and improving control measures [18].

Previous studies suggest that seasonal changes, influenced by factors like temperature and humidity, can affect bacterial infection rates [10]. Our study found significant seasonal variations in bacterial infections, with peaks in spring (March-April), as winter transitioned into summer and later months (October, November, and May). In contrast, the period from June to September had moderate infection rates. Lower rates were observed during winter (December-February), particularly in January, suggesting that environmental factors such as temperature and humidity may influence infection patterns. These findings are consistent with a previous study by Kumar et al., which reported higher infection rates in August and the lowest in December in India [19].

In this study, *S. aureus *was the most frequently isolated pathogen, followed by *K. pneumoniae*, *P. aeruginosa*, *A. baumannii*, *E. coli*, and *E. faecalis*. This pattern aligns with global data, where *S. aureus*, *E. coli*, *S. pneumoniae*, *K. pneumoniae*, and *P. aeruginosa* are commonly identified as leading pathogens [16]. Of particular note, *A. baumannii* infections were emerging as a concern. Heatmap analysis and time-series graphs demonstrated distinct seasonal trends: *S. aureus* infections peaked in June and October, while *E. faecalis* infections peaked in January and February, suggesting a possible relationship with seasonal factors. *P. aeruginosa* showed increased infection rates in January and November, indicating its environmental persistence during colder months. In contrast, *E. coli*, *A. baumannii*, and *K. pneumoniae* exhibited consistent infection rates year-round, possibly due to their adaptation to hospital environments. Comparatively, a study conducted in the United States observed that *Enterococcus *infections peak in spring, while *S. aureus* and *K. pneumoniae* peak in summer, and *E. coli *displayed no distinct seasonal pattern [20]. This suggests that regional environmental factors play a significant role in shaping the seasonality of bacterial infections.

Antibiotic resistance profiles revealed concerning trends. *S. aureus* demonstrated high resistance to beta-lactams, macrolides, and fluoroquinolones, with methicillin-resistant strains prevalent. In contrast, a 2021 study reported full susceptibility to trimethoprim/sulfamethoxazole and lower gentamicin resistance (40%) [21], while a study in Nepal found minimal gentamicin resistance and no resistance to vancomycin, indicating potential regional differences in resistance patterns [22]. The absence of resistance to last-resort antibiotics such as daptomycin, linezolid, and tigecycline provides treatment options but emphasizes the urgent need for adaptive infection control strategies, as Methicillin-resistant Staphylococcus aureus (MRSA) caused approximately 130,000 deaths globally in 2021 [23].

*K. pneumoniae* exhibited high resistance to carbapenems, fluoroquinolones, and aminoglycosides, with strains showing resistance to colistin and tigecycline, signaling the emergence of XDR strains. A previous study reported that 60% of *K. pneumoniae* isolates as XDR, 30% as MDR, and only 9% as susceptible, highlighting the rapid rise of carbapenem-resistant Enterobacteriaceae (CRE) [24]. This is consistent with global meta-analysis that reveals the growing threat of CRE, emphasizing the urgent need for novel antimicrobial strategies [23].

*P. aeruginosa* demonstrated significant resistance across multiple antibiotic classes, particularly beta-lactams, aminoglycosides, and fluoroquinolones, with resistance rates exceeding 50% for key drugs such as ceftazidime, ciprofloxacin, and levofloxacin. Alarmingly, 12% resistance to colistin with 88% intermediate resistance, highlights the increasing challenge of managing infections caused by this pathogen. In comparison, a previous study reported higher resistance rates, with 97% resistance to colistin, 75% to PTZ, 42% to cefepime, and 34% to ciprofloxacin [25].

*A. baumannii* showed extensive resistance to most antibiotic classes, including beta-lactams, fluoroquinolones, and aminoglycosides, complicating treatment options. Colistin remains a potential therapeutic option, but increasing intermediate resistance necessitates close monitoring. *E. coli* exhibited widespread resistance to several antibiotic classes, including beta-lactams and fluoroquinolones, complicating treatment. While colistin remains an intermediate option, tigecycline presents a promising therapeutic alternative for resistant strains. *E. faecalis* showed high resistance to macrolides, beta-lactams, and aminoglycosides but remained susceptible to last-resort antibiotics like linezolid, vancomycin, and daptomycin, ensuring effective treatment options. A meta-analysis found resistance rates of 60% for erythromycin, 49% for gentamicin, and under 2% for linezolid and daptomycin, emphasizing the need for ongoing surveillance to monitor rising resistance trends [26].

The study’s limitations include its single-center design, relatively small sample size, and focus on hospital-associated pathogens while excluding organisms like *Mycobacterium tuberculosis* and vector-borne bacteria, which may limit the generalizability of the findings. Future research should explore the mechanisms behind seasonal variations and antibiotic resistance, to improve infection control and treatment strategies.

## Conclusions

Our study highlights significant trends in bacterial infections, with peaks observed in spring (March-April), as well as alarming patterns of antibiotic resistance in hospital-associated pathogens. Seasonal peaks for pathogens like *S. aureus*, *P. aeruginosa*, and *E. faecalis *suggest a potential link between environmental factors and infection rates, while consistent year-round infections by *K. pneumoniae*, *E. coli*, and *A. baumannii* indicate their adaptability to hospital settings. These findings emphasize the importance of region-specific monitoring of infection dynamics to predict and manage outbreaks.

The rising prevalence of MDR organisms, particularly MRSA and CRE, emphasizes the urgent need for robust infection control measures and adaptive antibiotic stewardship programs. Resistance to critical antibiotics such as beta-lactams, fluoroquinolones, and carbapenems was particularly concerning. While susceptibility to last-resort antibiotics remains a critical lifeline, the emergence of intermediate resistance highlights the need for sustained surveillance and innovative antimicrobial strategies to combat the growing threat of antibiotic resistance.
